# Effects of Drying on the Properties and Applications of Alkaline Corn Cob Lignin

**DOI:** 10.1021/acsomega.6c06720

**Published:** 2026-10-17

**Authors:** Carlos Eduardo de Araújo Padilha, Jadna Lúcia de Freitas Silva, Francisco Bruno Ferreira de Freitas, Gleyson Batista de Oliveira, Augusto Monteiro de Souza, Darlisson de Alexandria Santos, Thelma Sley Pacheco Cellet, Renata Martins Braga, Emmanuel Damilano Dutra, Jackson Araújo de Oliveira, Domingos Fabiano de Santana Souza

**Affiliations:** † Laboratory of Alternative Energy and Transport Phenomena, Chemical Engineering Department, Federal University of Rio Grande do Norte (UFRN), Natal 59065-640, RN, Brazil; ‡ Laboratory of Biochemical Engineering, Chemical Engineering Department, Federal University of Rio Grande do Norte (UFRN), Natal 59078-970, RN, Brazil; § Department of Cell Biology and Genetics, Biosciences Center, Federal University of Rio Grande do Norte (UFRN), Natal 59078-970, RN, Brazil; ∥ Research Group on Biomass Energy, Department of Nuclear Energy, 28116 Federal University of Pernambuco (UFPE), Recife 50670-901, PE, Brazil; ⊥ Polymeric and Composite Materials Group, Department of Chemistry, State University of Maringá (UEM), Maringá 87020-900, PR, Brazil; # Enviromental Technology Laboratory, Department of Chemistry, Federal University of Rio Grande do Norte (UFRN), Natal 59078-970, RN, Brazil

## Abstract

Lignins are macromolecules obtained from plant biomass with great potential for high-value industrial applications. However, gaps remain regarding how lignin processing, particularly drying, affects its valorization. This study investigated how different drying techniques affect the physicochemical and biological properties of corn cob lignin, including color, UV-blocking ability, antioxidant activity, antimicrobial potential, and toxicity. Lignin was extracted via alkaline pretreatment, and the suspension was dried using four methods: freeze-drying (L), spouted bed at 60 °C (SB), oven at 60 °C (O60), and oven at 105 °C (O105). UV analysis confirmed that air exposure and temperature increases led to condensation reactions due to the loss of phenolic hydroxyl groups. Interestingly, spouted bed drying preserved the original color and maintained a phenolic hydroxyl content similar to freeze-drying, while exhibiting the highest antimicrobial activity against Gram-positive bacteria. Oven-dried lignins (O60 and O105) showed enhanced UV-blocking capabilities and superior *in vitro* DPPH radical scavenging but displayed lower ABTS inhibition efficiency (∼0.02 mg/mL vs 0.012 mg/mL for L). No correlation was observed between the lignin drying method and the zebrafish survival rate within the studied range (up to 100 mg/L). These findings demonstrate that drying techniques can strategically modulate lignin functional properties, serving as a crucial processing step for developing macromolecular functional products.

## Graphical abstract

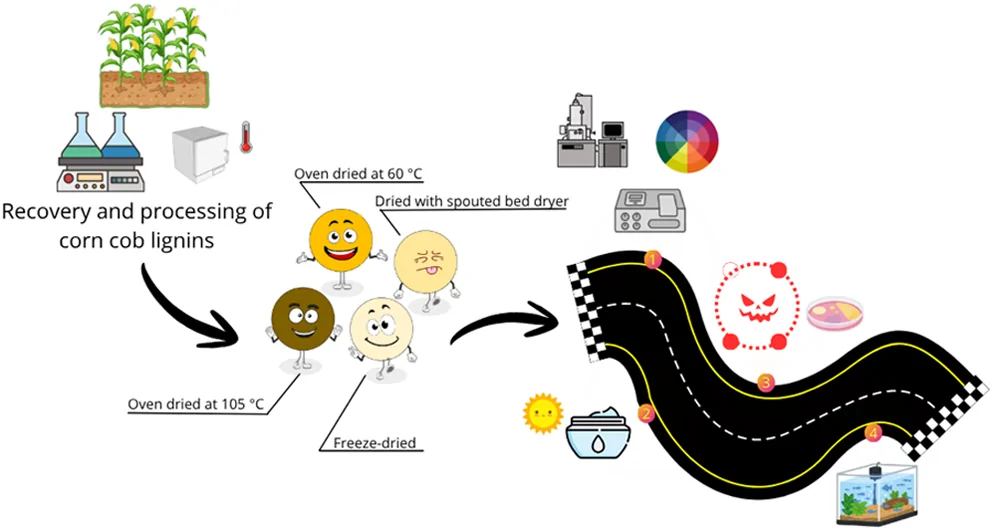

## 1 Introduction

The transition toward a circular bioeconomy has driven the replacement of fossil-based polymers with renewable resources, aiming to mitigate climate change.^1^ In this context, lignin is the most abundant natural aromatic polymer, accounting for up to 35% of lignocellulosic biomass.^2,3^ From a chemical standpoint, lignin is a complex three-dimensional macromolecule composed of phenylpropanoid units (*p*-coumaryl, coniferyl, and sinapyl alcohols) linked by ether and carbon–carbon bonds, featuring a high density of carboxylic acids and hydroxyl groups (both phenolic and aliphatic).^1^

Despite its potential as a precursor to high-value-added materials such as phenolic resins,^4^ functional foams,^5^ and carbon fibers,^6^ approximately 98% of lignin still undergoes energy valorization through direct combustion. This utilization route stems, in part, from the structural heterogeneity imposed by isolation methods.^7^ While alkaline routes offer advantages over Kraft processes by yielding sulfur-free polymers with reduced coloration and higher reactivity, lignin’s macromolecular integrity remains highly sensitive to postprocessing stages, particularly drying.^8^

Drying is a critical unit operation, often overlooked in isolation protocols, yet it can induce irreversible structural transformations, such as reducing active water sorption sites.^9,10^ Although freeze-drying preserves the original morphology due to low temperatures, its limited scalability drives the use of conventional thermal drying.^11^ However, prolonged heat exposure in forced-air ovens (above 60 °C) promotes condensation reactions, increases molecular weight, reduces phenolic hydroxyl content, and promotes oxidation.^11,12^ Consequently, high-rate drying processes that minimize thermal stress are needed. Spray drying has been successfully employed at an industrial scale to produce lignin-based dispersants because it avoids thermal degradation of the macromolecule; however, it requires a large initial investment.^13^ Therefore, the spouted bed emerges as a promising alternative, operating through the cyclic contact of suspensions with inert particles under high gas flow, which ensures high heat transfer rates and short residence times, similar to atomization.^14,15^ During spouted bed drying, the feed material coats the moving hot particles, enabling rapid water evaporation and the detachment of dry powder through attrition, which is subsequently collected via a cyclone. Although spouted bed technology is well established at large scale and used for heat-sensitive materials,^16^ it has never been applied to lignin drying; consequently, its effects on lignin’s macromolecular architecture and functional properties remain unknown. The unique characteristics of the spouted bed suggest it may preserve the phenolic hydroxyl groups of lignins, yielding results comparable to freeze-drying.

In this study, we investigate how different drying techniques (freeze-drying, oven drying, and spouted bed drying) modulate the physicochemical characteristics and biological performance of alkaline lignin extracted from corn cobs. Using corn cob as a feedstock is justified by its production of low-pigmentation lignin, allowing more refined spectroscopic and visual analysis of the alterations induced by thermal processing. Furthermore, this study aimed to go beyond conventional lignin structural elucidation and explore a wider range of properties that could expand the use of this biomolecule. We evaluated the preservation of the polyphenolic structure and its direct correlation with UV-blocking capacity, antioxidant activity, antimicrobial potential, and *in vivo* toxicity.

## 2 Materials and Methods

### 2.1 Collection and Processing of Corn Cobs

Corn cob samples were collected from the rural area of Simão Dias (10° 44′ 20″ S, 37° 48′ 36″ W), located in Sergipe, Northeast Brazil. The corn cobs were milled using a TRE25 forage chopper (Tramontina, Brazil) and sieved through a 20-mesh sieve. The corn cob powder was stored in plastic containers and kept in a dry place away from light.

### 2.2 Chemicals

Sulfuric acid, ethanol, methanol, potassium persulfate, and sodium hydroxide were purchased from Synth (São Paulo, Brazil). 2,2-diphenyl-1-picrylhydrazyl (DPPH), 2,2′-azinobis (3-ethylbenzothiazoline-6-sulfonic acid) (ABTS), and 1% dimethyl sulfoxide (DMSO) were purchased from Sigma-Aldrich (USA); nutrient broth and Baird-Parker agar were purchased from Sigma-Aldrich. NIVEA Soft moisturizing cream was purchased locally.

### 2.3 Lignin Recovery

Alkaline pretreatment with sodium hydroxide was employed to extract lignin from the corn cob. Using 4 L beakers as reactors, 300 g of corn cob was mixed with a 2% (w/v) sodium hydroxide solution using a glass rod, and the beakers were placed in a thermostatic bath at 80 °C. After 1 h of reaction, the alkaline hydrolysate was collected, filtered through a cloth filter, and subsequently subjected to vacuum filtration. The filtrate was then acidified with concentrated sulfuric acid until pH 2.0, where a change in color and rheology was observed due to lignin precipitation. The lignin was recovered by centrifugation using an SL700 centrifuge (Solab, Brazil) at 3,500*g*, followed by washing with acidified water (pH 2.0, adjusted with sulfuric acid). The resulting lignin was stored in a refrigerator at 7.5% (w/v) solids for subsequent drying experiments.

### 2.4 Lignin Drying

The freeze-drying was initiated by transferring the lignin suspension to conical tubes and placing them in the manifolds of an LS3000 freeze-dryer (Terroni, Brazil), which operated at −80 °C and 100 mTorr for 72 h. The freeze-dried corn cob lignin was coded as lignin “L”.

Spouted bed drying was performed in a homemade apparatus, which consists of: (i) blower, (ii) exhaust and passage valves, (iii) heat exchanger, (iv) thermocouples, (v) glass column, and (vi) cyclone (Figure 1). Using 100 g of high-density polyethylene (HDPE) as inert particles, the drying was conducted by feeding 400 mL of the lignin suspension (maintained under magnetic stirring) at an air velocity of 55 m/s and a temperature of 60 °C. The dried lignin after the procedure was collected at the cyclone outlet and coded as lignin “SB”.

**Figure 1:**
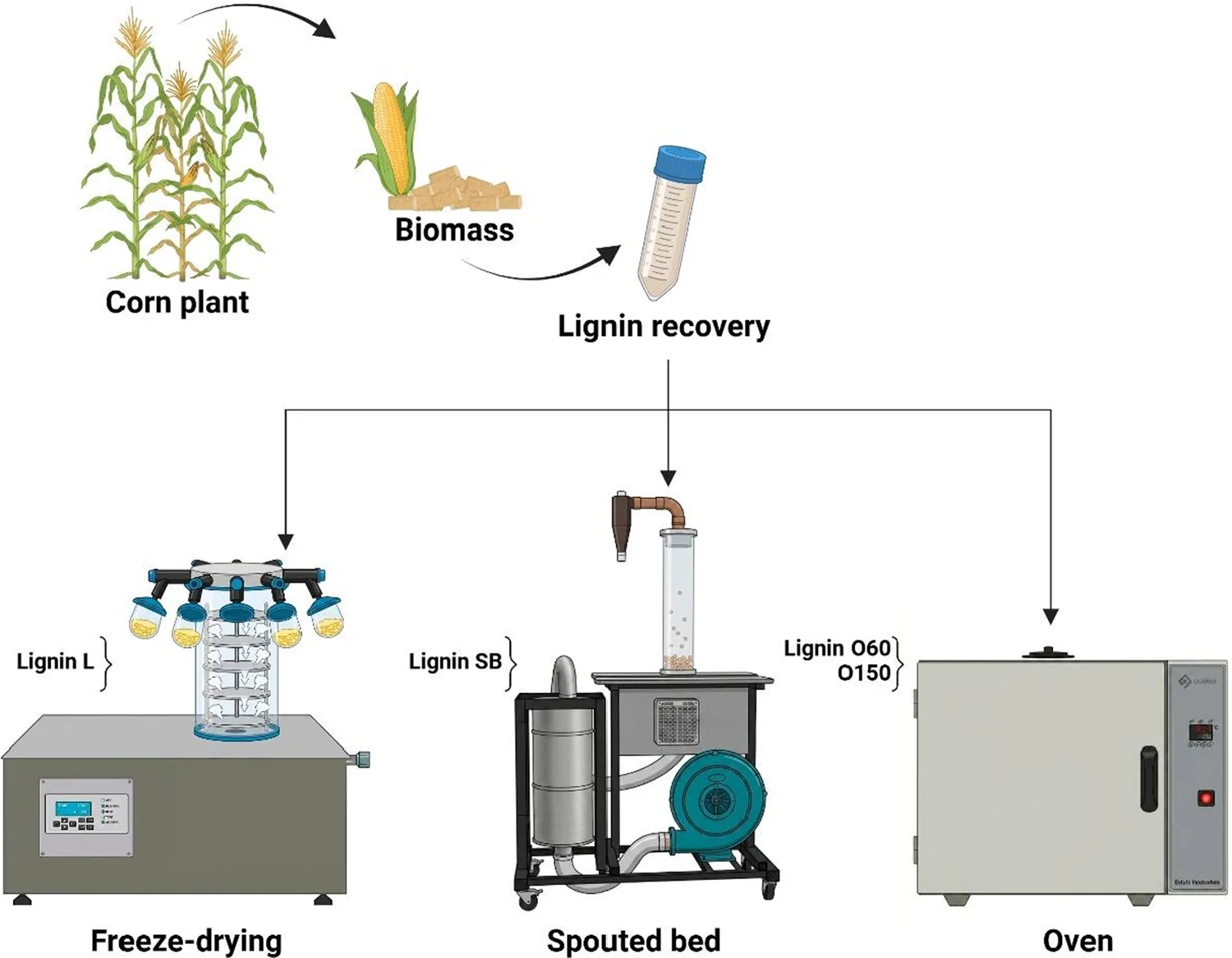
Schematic diagram of lignin drying systems. The images are adaptations of BioRender templates (2026). Retrieved from https://app.biorender.com/biorender-templates.

Lignins dried in a forced-air oven were processed in a Q314 M oven (Quimis, Brazil) at temperatures of 60 and 105 °C for 24 h. In each condition, the lignin suspension was transferred to polyethylene trays until it formed a liquid film 1 cm thick. The lignins dried in the oven at 60 and 105 °C were coded as “O60” and “O105”, respectively. The dry lignin yield, lignin moisture, and energy expenditure of each drying process were evaluated.

### 2.5 Determining Moisture and Color Parameters

The moisture content values of lignins L, SB, O60, and O105 were determined by the difference in mass after incubation at 105 °C for 24 h. The color parameter measurements of lignins L, SB, O60, and O105 were performed using a GOYOJO portable digital colorimeter. The L*, a*, and b* parameters presented were generated from the average of eight measurements. The color difference between the samples was calculated using eq 1, with lignin “L” as the target.

1 $$\Delta E=\sqrt{{\left(L_{t}^{*}-L_{sample}^{*}\right)}^{2}+{\left(a_{t}^{*}-a_{sample}^{*}\right)}^{2}+{\left(b_{t}^{*}-b_{sample}^{*}\right)}^{2}}$$

### 2.6 UV Profile and Phenolic Hydroxyl Content

Effects of the drying methods on corn cob lignin in absorbance measurements in the UV region using a Genesys 10 UV/vis spectrophotometer (ThermoSpectronic, USA). Samples were prepared by dissolving 20 mg of lignin (L, SB, O60, and O105) in 10 mL of ethanol under magnetic stirring. The scan was performed in the 280–400 nm range. The contents of C5-condensed, noncondensed, conjugated, and nonconjugated hydroxyl groups were calculated using the methodology of Gärtner et al.^17^

### 2.7 Thermogravimetric Analysis (TGA), Fourier-Transform Infrared Spectroscopy (FTIR), and Transmission Electron Microscopy (TEM)

TGA profiles were obtained in an SDT Q600 instrument (TA Instruments, France). The equipment was operated from 25 to 600 °C, with a sample mass of 40 mg, a heating rate of 25 °C/min, and an inert environment (synthetic air at 250 mL/min). The FTIR profiles (transmittance) of the dried lignins were analyzed on a Tensor-II spectrometer (Bruker, USA) over 4000 to 600 cm^–1^ at a resolution of 1 cm^–1^. Transmittance values were converted to absorbance, and two semiquantitative metrics were measured: the Condensation Index (CI) and the absorbance ratio in the 1510 and 1460 cm^–1^ regions. Based on Faix et al.,^18^ CI values are obtained using eq 2.

2 $$CI=\frac{\sum \mathrm{Al}l\;absorbance\;\min ina\;between\;1500\;and\;1050\;{cm}^{-1}}{\sum All\;absorbance\;\max ima\;between\;1600\;and\;1030\;{cm}^{-1}}$$

TEM analysis of lignins L, SB, O60, and O105 was performed using a JEM 1400 microscope (JEOL, Japan) at 80 kV. For sample preparation, dried lignin samples were dispersed in ethanol at 1 mg/mL and homogenized in a 3 L benchtop ultrasonic bath (Eco-Sonics, model Q3.0/40, 40 kHz) for 10 min. Subsequently, three 10 μL aliquots of each dispersion were deposited onto 400-mesh copper grids coated with a Formvar/carbon support film (FCF400-Cu, Electron Microscopy Sciences) by drop-casting using a Proline Plus micropipette (Sartorius). The grids were dried at room temperature for 24 h before TEM examination. No staining or additional contrast agent was used, and no cryogenic preparation was employed.

### 2.8 NMR Analysis

^1^H and ^13^C nuclear magnetic resonance (NMR) spectra of lignin samples were performed on a Varian URMNS 400 MHz spectrometer (Varian, USA) using DMSO-*d*_6_ as the solvent.

### 2.9 UV Profile and SPF of Moisturizing Cream Blended with Dried Lignins

To evaluate the SPF-enhancing effect, a commercial moisturizing cream was mixed with dried corn cob lignins in mass ratios of 99:1 and 98:2 (w/w, %) using a glass rod. The blends were spread on quartz surfaces at 2 mg/cm^2^ and left to stand for 20 min. Tests were performed in sextuplicate, and responses were recorded as transmittance using a Genesys 10UV/Vis spectrophotometer (ThermoSpectronic, USA). SPF values were calculated from the following eq 3

3 $$SPF=\frac{\sum_{290nm}^{400nm}E_{\lambda }S_{\lambda }}{\sum_{290nm}^{400nm}E_{\lambda }S_{\lambda }T_{\lambda }}$$

Where *E*_λ_ is the erythermal spectral effectiveness, *S*_λ_ is the solar irradiance spectral efficiency, and *T*_λ_ is the spectral transmittance of the sample. The values of *E*_λ_ and *S*_λ_ in the 290–400 nm range were obtained from the International Commission on Non-Ionizing Radiation Protection.^19^

### 2.10 In Vitro Antioxidant Activity

DPPH and ABTS radical scavenging analyses were conducted to evaluate the antioxidant potential of corn cob lignins (L, SB, O60, and O105). The DPPH analysis was performed by adding 1.00 mL of 0.06 mM DPPH (dissolved in ethanol) to cuvettes containing 200 μL of ethanolic lignin solutions (in triplicate), following the methodology of Brand-Williams et al.^20^ After agitation and 25 min of rest, readings were taken on a spectrophotometer at 517 nm. The ABTS analysis was performed by mixing 200 μL of lignin solutions (in ethanol) and 1300 μL of ABTS solution in cuvettes (in triplicate), according to Miller et al.^21^ After a 6 min incubation, measurements were taken on a spectrophotometer at 734 nm. The results were expressed as the percentage of free radical scavenging.

### 2.11 Antimicrobial Activity

The antibacterial capacity of dried corn cob lignins was evaluated against two bacterial strains: *Pseudomonas aeruginosa* ATCC 27853 (Gram-negative) and *Rhodococcus rhodochrous* NRRL B–2149 (Gram-positive). Antibacterial activity was examined using the agar disc diffusion method, following to the methodology described by Nasrollahzadeh et al.^22^ Sterile nutrient agar was placed in Petri dishes and incubated at 37 °C for 24 h. Bacterial suspensions at 1.5 × 10^8^ CFU/mL were spread uniformly on the agar surface. Each sterile disc was then dipped into the lignin suspensions with DMSO, and the discs were carefully placed on agar plates at least 2 cm apart (in triplicate). Discs containing only DMSO served as a negative control. The inoculated Petri dishes were incubated for 24 h at 37 °C. The diameter of each inhibition zone was used to measure the antibacterial activity.

### 2.12 Acute Lignin Toxicity Test in Zebrafish

#### 2.12.1 Animals and Housing

Acute toxicity assays were performed using zebrafish (*Danio rerio*) embryos of the wild-type AB strain. The maintenance of adult animals and experimental procedures were conducted in accordance with CONCEA Normative Resolution No. 34. The Ethics Committee previously approved all protocols on the Use of Animals of the Federal University of Rio Grande do Norte-CEUA/UFRN, under institutional certificate No. 329.006/2023. The health and well-being of the animals were monitored daily throughout all experimental phases.

Adult zebrafish were kept in the Zebrafish Laboratory (FishLab), Department of Physiology and Behavior, UFRN, in an automated water recirculation system, under controlled conditions of temperature, photoperiod, and water quality. The animals were kept in automated tanks with water at 27 ± 1 °C, pH 7.2, dissolved oxygen around 8.4 mg/L, total ammonia less than 0.01 mg/L, and an approximate conductivity of 600 μS/cm. The photoperiod was maintained in a 14 h light and 10 h dark cycle, with the light phase beginning at 6 h. Feeding was carried out three times a day with *Artemia sp*. nauplii and commercial feed.

#### 2.12.2 Obtaining Embryos

To obtain embryos, four breeding aquariums were used, each containing three male and two female zebrafish. The animals remained separated during the night and, after the start of the light phase, the barrier to reproduction was removed. The embryos were collected approximately 1 h after the lights were turned on. Next, viable eggs were selected using a stereomicroscope, with normal embryonic development up to the blastula stage within 1.5 h postfertilization (hpf) as the viability criterion. The entire procedure for obtaining and selecting embryos followed OECD protocol no. 236 (Fish Embryo Acute Toxicity Test).

#### 2.12.3 Embryos/Larvae Exposure to Lignin

Dried lignin samples were dissolved in 1% DMSO to prepare stock solutions, which were tested at concentrations of 6.25, 12.5, 25, 50, and 100 mg/L. The maximum concentration of 100 mg/L was established based on the recommendations of OECD protocol no. 236. System water served as the negative control, and 0.1% DMSO as the vehicle control. A solution of 3,4-dichloroaniline (3,4-DCA; 4 mg/L) was used as the positive control to confirm assay responsiveness.

The assay was conducted in 24-well polystyrene plates, following OECD protocol no. 236. Viable embryos up to 3 hpf were transferred individually to the wells, with one embryo per well. Two experimental replicates were performed. For each lignin concentration, the vehicle control, and the positive control, 20 embryos were evaluated per replicate, resulting in a total of 40 embryos per experimental condition. The negative control included 24 embryos per replicate, for a total of 48 embryos. The exposure was maintained for 96 h under controlled temperature and photoperiod conditions. Embryo evaluation was performed every 24 h up to 96 hpf using a stereomicroscope. During the exposure period, the main criteria for embryonic lethality were recorded, including egg coagulation, absence of somite formation, absence of tail detachment, absence of heartbeat, and embryonic or larval death. In addition to survival, phenotypic alterations associated with embryonic and larval development were evaluated.

Hatching was assessed at 48, 72, and 96 hpf and recorded cumulatively for each treatment and control group. For calculation of cumulative hatching rates, embryos that had coagulated during the first 24 h of exposure were excluded from the denominator because they were no longer eligible to hatch. Therefore, the number of embryos eligible for hatching was defined as the initial number of embryos minus the number of embryos coagulated at 24 h. Cumulative hatching rates were expressed as the number of hatched embryos relative to the number of embryos eligible for hatching, *n*/*N* (%).

### 2.13 Statistical Analysis

The results were expressed as arithmetic means and standard deviations. Mean comparison tests were performed to evaluate the significance of drying effects using one-way ANOVA with Tukey’s posthoc test in Statistica 7.0 software. Fisher’s exact tests were also performed in R version 4.6.0 (2026–04–24/R Core Team).

## 3 Results and Discussion

### 3.1 Drying Parameters

Overall, all drying methods effectively removed water from the lignin samples, reducing moisture content from an initial 7.5% to as low as 0% in the forced-air oven test at 105 °C. Temperatures of 100–105 °C are commonly used for moisture determination analyses, and alkali lignin derived from corn cobs is not a hygroscopic material. The analysis was also performed immediately after sample collection.

The lignin obtained via freeze-drying had the highest moisture content (6.3%), which also contributed to a higher yield than the other methods. This value aligns with literature findings reported by Ponnudurai et al.^23^ and Bhadane et al.^24^ The sample might have required more time to reduce moisture further, but this would have entailed even higher energy consumption, which was already 61.2 kWh. Oven drying was also energy-intensive, consuming 7.8 kWh and 14.4 kWh at 60 and 105 °C, respectively.

Spouted bed drying met expectations and demonstrated the lowest energy consumption, as the process (despite operating at 60 °C) lasted less than 8 h. Although it involves operating a blower and a heater, the total energy consumption of 5.4 kWh is more than ten times lower than that of freeze-drying. A drawback of the technique was lignin loss during the process. In contrast, oven drying retains the entire lignin mass; however, a significant portion of the product was lost due to adsorption onto the HDPE spheres, a process that also required periodic air-velocity adjustments to maintain the bed regime. Although the cyclone used in the spouted bed apparatus was designed to collect micrometer-sized samples, significant quantities of lignin were also lost, escaping with the airflow (Table 1).

**Table 1:** Parameters Associated with the Drying of Corn Cob Lignin

Drying method	Yield (%)	Moisture content (%)	Energy consumption (kWh)
Freeze-drying	8.0	6.3	61.2
Spouted bed drying	1.1	7.6	5.4
Oven drying–60 °C	7.8	6.6	7.8
Oven drying–105 °C	7.5	0	14.4

### 3.2 Color

Drying intensity strongly affects the color of corn cob lignin. While lignins L and SB exhibited a yellowish coloration, O60 and O105 showed light brown and dark brown tones, respectively (Figure 2).

**Figure 2:**
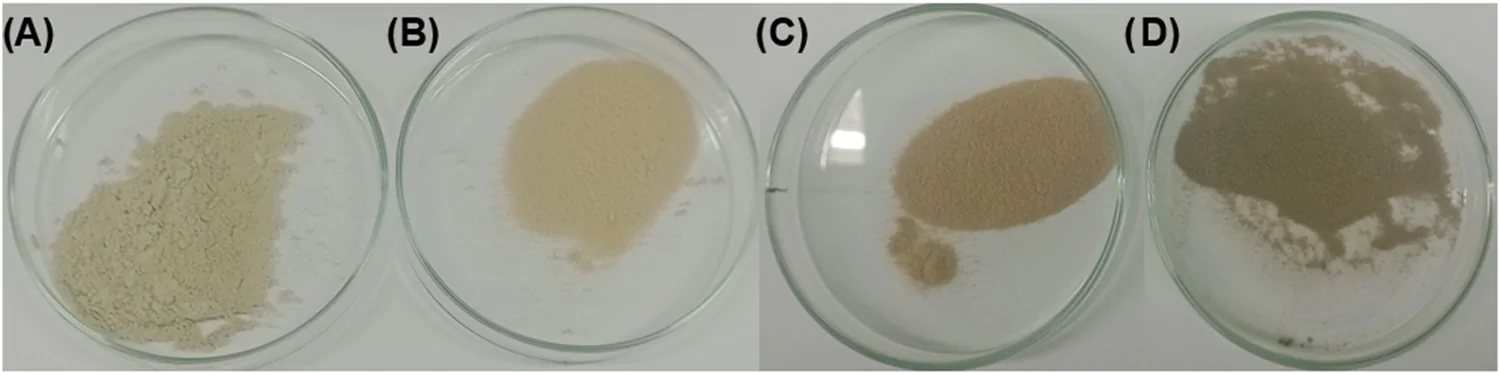
Images of corn cob lignins dried by freeze-drying (L-A), spouted bed (SB-B), and air circulation oven at 60 °C (O60–C) and 105 °C (O105-D). Source: Own authorship.

In quantitative terms, the Δ*E* values ranged from 3.23 for lignin SB to 10.19 for O105, as shown in Table 2. In fact, the lignin SB appeared visually more similar to lignin L due to the L* (luminosity) and a* (redness) parameters, resulting in a Δ*E* of 3.23. Although it also involves heating to remove water, lignin residence time is low in the spouted bed dryer, implying less stress.^25^ This behavior has also been observed in other studies evaluating lignin drying.^11,26^ The following subsections discuss lignin structure in more detail.

**Table 2:** Parameters Relating to the Coloration of Corn Cob Lignin Ssamples Dried by Freeze-Drying (L), Spouted Bed (SB), and Air Circulation Oven at 60 °C (O60) and 105 °C (O105)^a^

Samples	L*	a*	b*	Δ*E*
L	23.18 ± 1.19^a^	–0.17 ± 0.05^d^	11.89 ± 0.73^bc^	-
SB	23.73 ± 2.54^a^	2.62 ± 0.22^b^	13.42 ± 0.54^b^	3.23
O60	26.10 ± 1.04^a^	3.57 ± 0.12^a^	17.06 ± 0.14^a^	7.02
O105	13.30 ± 0.72^b^	1.65 ± 0.03^c^	10.14 ± 0.64^c^	10.19

a Lowercase letters indicate no statistical difference between the parameters at the *p* < 0.05 level.

### 3.3 Lignin Morphology by TEM

TEM analysis showed that the drying method impacts the shape of the lignin structures in corn cob. After isolation from alkaline pretreatment, the lignins in the initial suspension should already present as nanometric structures due to self-assembly,^27,28^ as observed in the images in Figure 3. Both samples obtained by freeze-drying and by spouted bed drying presented particles with greater dispersivity and a spherical shape on the order of 50–100 nm. According to Österberg et al.,^29^ the spherical shape of lignins is particularly desirable for technological applications because it offers better reproducibility and is more efficient as a colloid. In contrast, samples obtained by air-circulation drying (O60 and O105) showed irregularly shaped, heterogeneous nanoparticles with high agglomeration. Because of this characteristic, these lignins may be more difficult to disperse and solubilize. The higher particle density may also cause lignin to appear darker on a macroscopic scale.

**Figure 3:**
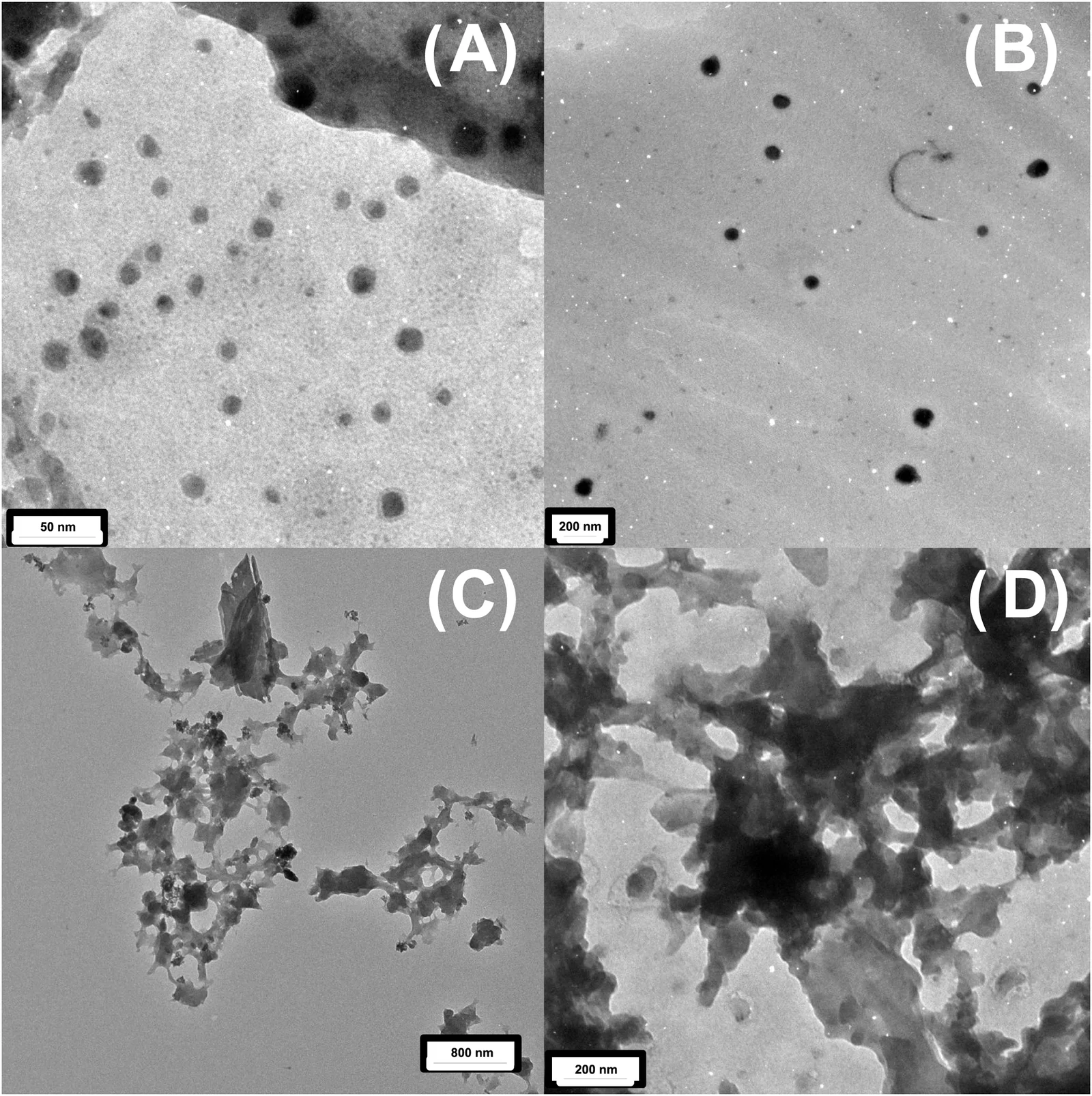
TEM images of corn cob lignins dried by freeze-drying (L-A), spouted bed drying (SB-B), and air circulation oven at 60 °C (O60–C) and 105 °C (O105-D).

### 3.4 UV Profile and Phenolic Hydroxyl Content

UV blocking is one of the most praised properties of lignins, as confirmed by the absorbance scanning analysis shown in Figure 4. All lignin samples showed high absorption values in the UVB region (>1.20), which decreased sharply to 0.05 at 400 nm. Lignins are phenylpropanoid molecules, and their alternating double bonds function as chromophores, primarily by promoting electron transitions from lower-energy orbitals to higher-energy orbitals.^30^

**Figure 4:**
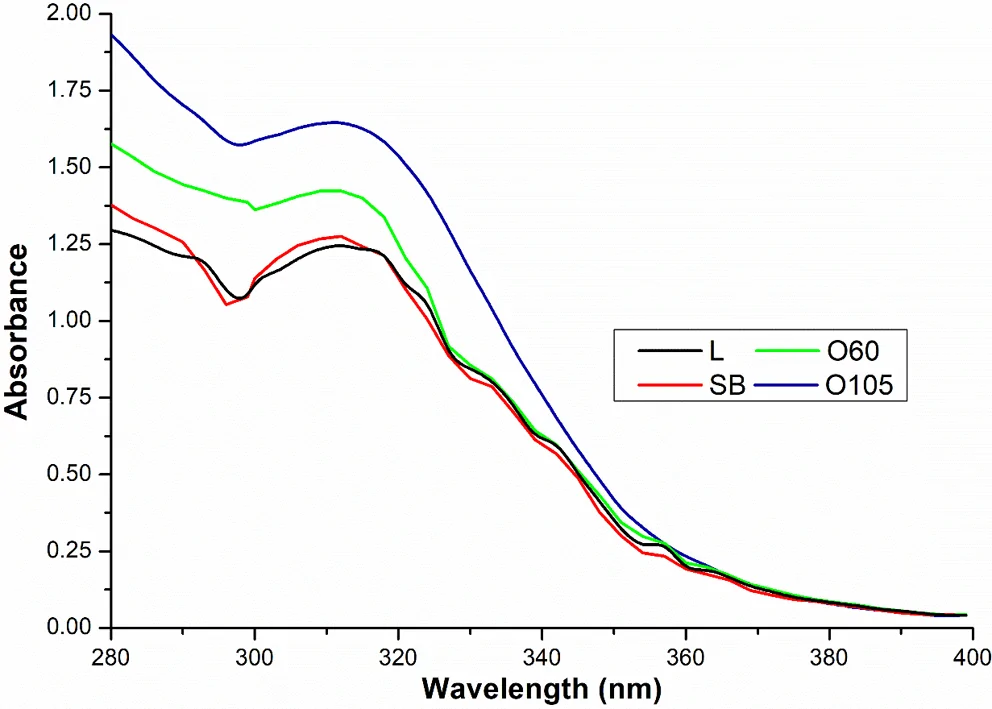
Absorbance of ethanolic solutions of corn cob lignins dried by freeze-drying (L), spouted bed (SB), and air circulation oven at 60 °C (O60) and 105 °C (O105). The lignin concentration was fixed at 20 mg/L.

The phenolic hydroxyl group is a good indicator of macromolecular degradation and can be quantified from the UV profile. Lignins L and SB experienced less heat stress and showed higher phenolic hydroxyl values than the oven-dried samples (O60 and O105) in all configurations (Table 3). The availability of noncondensed phenolic hydroxyl groups drives lignin reactivity. The value of 0.4 obtained for L and SB is low, yet it falls within the same order of magnitude as other reports on agricultural residues.^31,32^ The total phenolic hydroxyl content also agrees with the color results, showing the following sequence: L (1.02 mmol/g) > SB (0.95 mmol/g) > O60 (0.77 mmol/g) > O105 (0.74 mmol/g).

**Table 3:** Content of Phenolic Hydroxyls in Lignin Samples Dried by Freeze-Drying (L), Spouted Bed (SB), and Air Circulation Oven at 60 °C (O60) and 105 °C (O105)^a^

Functional group	L	SB	O60	O105
C5-Condensed (mmol/g)	0.11 ± 0.01^a^	0.06 ± 0.00^b^	0.06 ± 0.00^b^	0.04 ± 0.00^c^
Uncondensed (mmol/g)	0.40 ± 0.02^a^	0.40 ± 0.01^a^	0.31 ± 0.01^b^	0.32 ± 0.01^b^
Conjugated (mmol/g)	0.16 ± 0.01^ab^	0.19 ± 0.01^a^	0.15 ± 0.01^b^	0.15 ± 0.00^b^
Unconjugated (mmol/g)	0.35 ± 0.03^a^	0.30 ± 0.02^ab^	0.24 ± 0.02^ab^	0.22 ± 0.01^b^
Total (mmol/g)	1.02 ± 0.05^a^	0.95 ± 0.03^ab^	0.77 ± 0.03^bc^	0.74 ± 0.02^c^

a Lowercase letters indicate no statistical difference between the parameters at the *p* < 0.05 level.

Although this result aligns with the logic of interaction with the drying process, it is important to note that phenolic hydroxyls act as auxochromes, donating electrons to the aromatic rings and theoretically enhancing UV-blocking capacity.^33^ Therefore, the higher absorbance values observed in lignins O60 and O105 must involve other mechanisms. One hypothesis is that oven drying removes side-chain groups and triggers condensation reactions, resulting in a more compact molecular structure that facilitates π-stacking interactions and enhances UV-photon-induced electronic transitions.^34^ Another hypothesis, which counters the phenolic hydroxyl content argument, involves the formation of new chromophores. Quinone groups can form through the breakdown of phenolic hydroxyls upon exposure to oxygen and high temperatures; these groups strongly absorb UV radiation due to their cyclic diketone structure featuring conjugated π systems.^35,36^

### 3.5 TGA, FTIR, and NMR

The thermogravimetric degradation profile of lignins exhibited two well-defined events (Figure 5). The first event is due to moisture loss when the ambient temperature approaches 100 °C. The moisture loss recorded in O105 is due to water absorption during sample storage. The other degradation event occurred when the temperature approached 200 °C, which is relatively low for isolated lignin. This behavior can be attributed to the nature of corn cob lignin, which generally has a higher S/G ratio (>1.0), a more open structure, and greater susceptibility to thermal degradation.^37^ The DTG peaks of the lignins in this second event are generally close, in the range of 200–210 °C. After this, all lignin samples showed monotonic behavior up to 600 °C.

**Figure 5:**
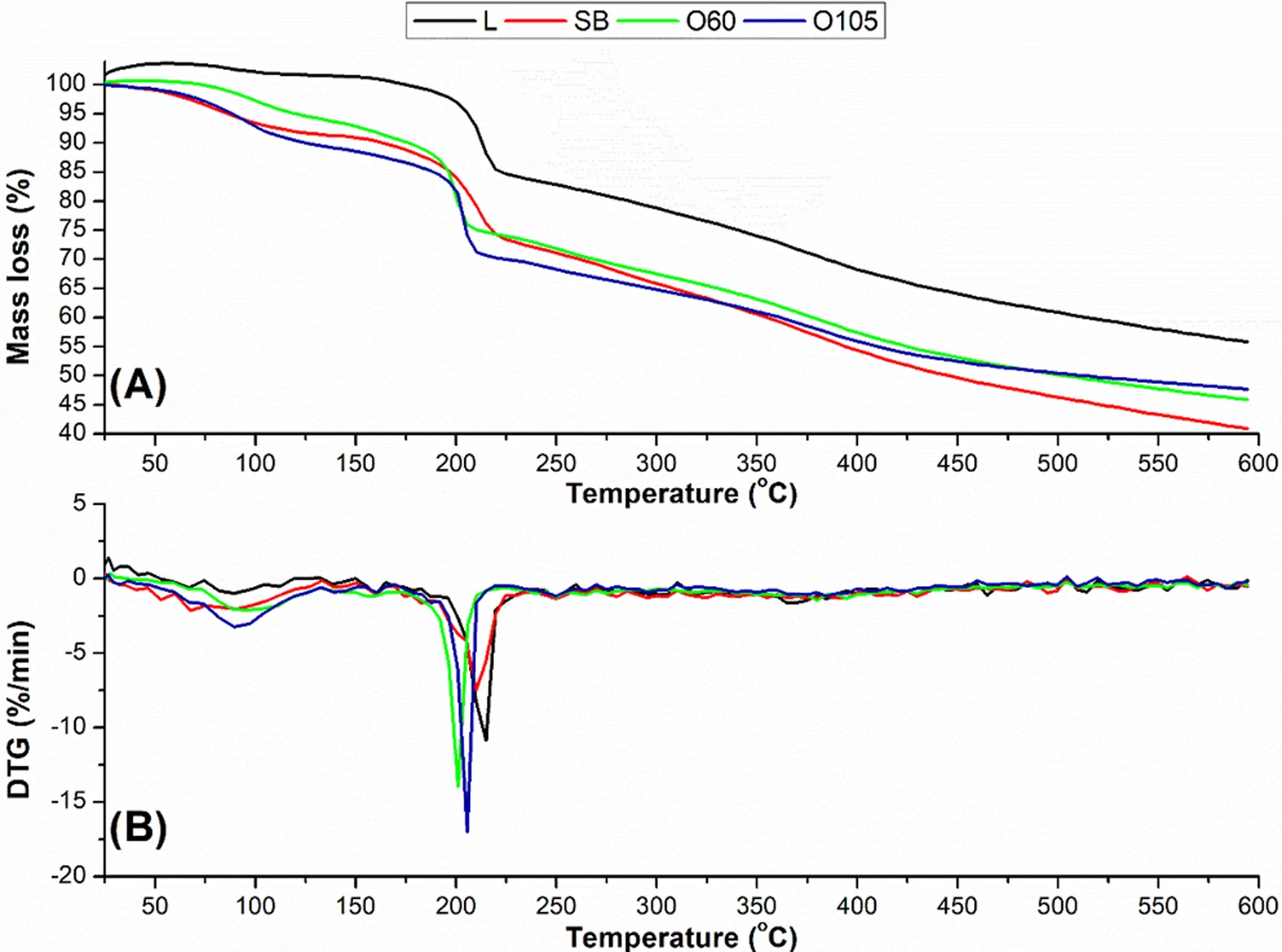
Mass loss (A) and DTG (B) during thermal degradation of corn cob lignins dried by freeze-drying (L), spouted bed (SB), and air circulation oven at 60 °C (O60) and 105 °C (O105).

In general, the FTIR spectra of all samples show similar behavior, but some differences were still observed, as shown in Figure 6. The band related to C–O bonds of tertiary alcohol functions at 1159 cm^–1^ is present in samples L and SB but absent in samples O60 and O105, corroborating the loss of hydroxyl groups due to heating. The broad band related to O–H stretching in lignin L was centered at 3357 cm^–1^ but was shifted to higher wavenumbers in lignins O60 (3372 cm^–1^) and O105 (3366 cm^–1^). Sample O60 also exhibited some distinctive features: there was a reduction in the intensity of the O–H stretching band at 1039 cm^–1^, while the out-of-plane and O–H stretching bands at 1090 and 609 cm^–1^ were more clearly defined.^38^ The latter observation indicates the formation of a new condensed ether or hemiacetal structure, according to Ghahri and Park.^39^

**Figure 6:**
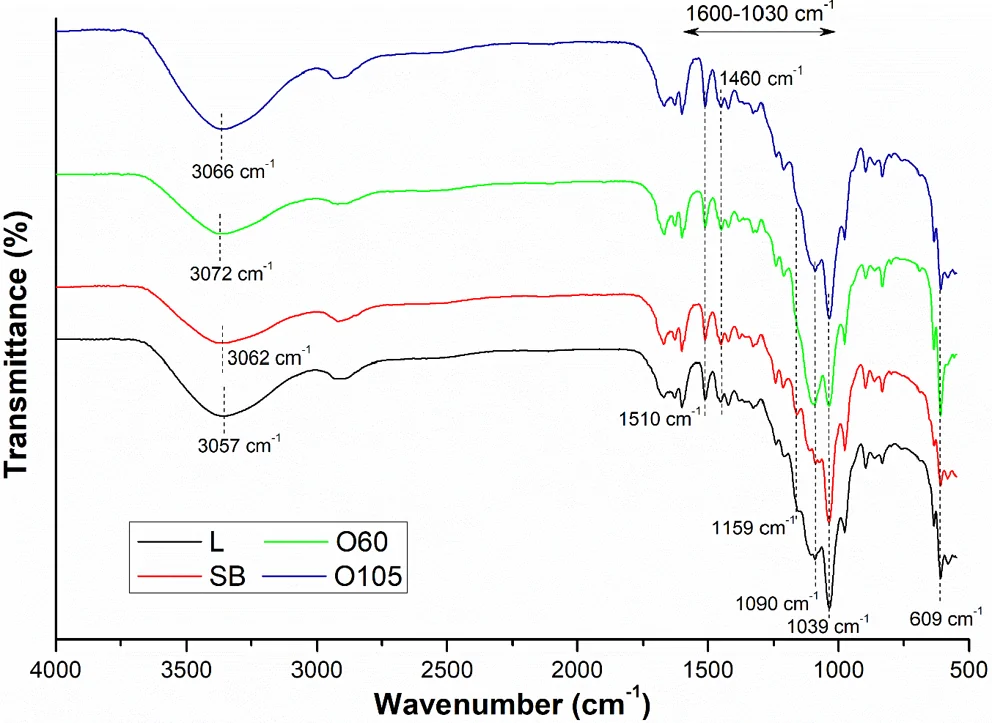
FTIR profiles of corn cob lignins dried by freeze-drying (L), spouted bed (SB), and air-circulation oven at 60 °C (O60) and 105 °C (O105).

Although the FTIR profiles did not show notable differences in the disappearance of O–H bands and the appearance of C–C bands, the data in Table 4 support structural changes in the macromolecule. The band at 1460 cm^–1^ corresponds to the asymmetric deformation of C–H bonds in methyl and methylene groups of side-chain moieties, whereas the band at 1510 cm^–1^ serves as a stable reference for the aromatic skeleton. Consequently, changes in the *A*_1460_/*A*_1510_ ratio suggest the loss of groups within the side-chain structure;^40^ this likely occurred in samples O60 and O105, as they exhibited the lowest values for this metric (0.83 and 0.92). Lignin L showed the lowest CI value among all samples (0.27), while lignin O105 showed the highest value (0.54). According to Faix,^18^ harsher processing methods yield higher minimum and maximum values across the FTIR profile, reflected in elevated CI values approaching 1.0. In Figure S1, the CI values also show a strong correlation with Δ*E* data (*R* ^2^ = 0.903) and total phenolic content determined via UV profile (*R* ^2^ = 0.734).

**Table 4:** Condensation Index (CI) Values and *A*_1460_/*A*_1510_ Absorbance Ratios Calculated from FTIR Profiles of Lignin Samples Dried by Freeze-Drying (L), Spouted Bed (SB), and Air-Circulation Oven at 60 °C (O60) and 105 °C (O105)

FTIR metric	L	SB	O60	O105
CI	0.27	0.36	0.38	0.54
*A*_1460_/*A*_1510_	0.98	0.94	0.83	0.92

Figure 7A shows the ^1^H NMR spectrum results. In all spectra of the four lignin samples, water protons appear between 3.0 and 4.0 ppm. The signals at 6.7–6.8 and 7.0 ppm are attributed to the aromatic protons of the S and G units of lignin.^41^ These peaks are less evident in sample O105, possibly because these groups were lost during the slightly higher-temperature application. The H unit, as well as hydroxycinnamic acids in the studied lignins, can be confirmed by signals between 7.2 and 7.5 ppm.^42^ These signals are more evident in the lignin SB.

**Figure 7:**
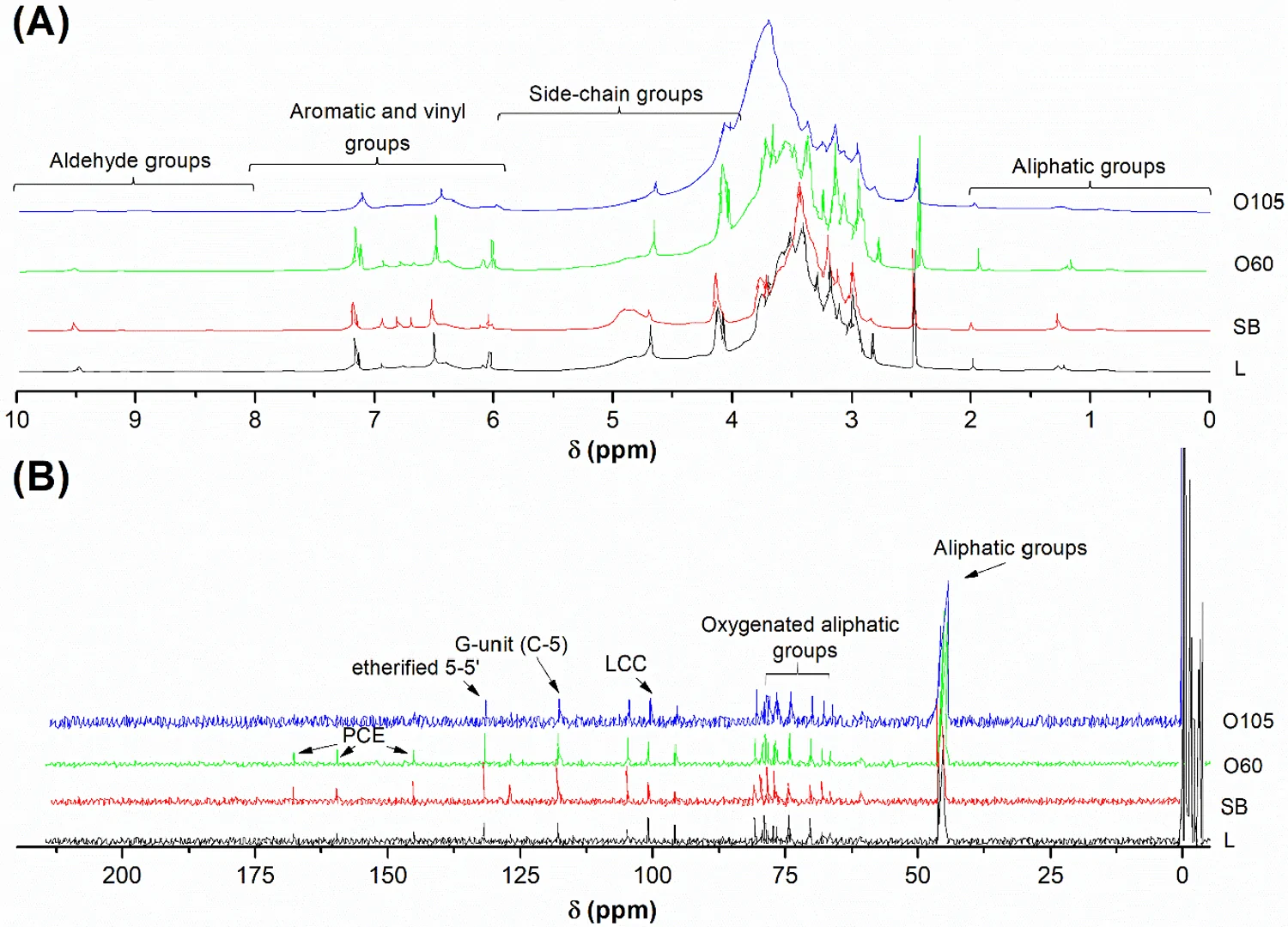
^1^H NMR (A) and ^13^C NMR spectra (B) of corn cob lignins obtained via different drying methods, being freeze-drying (L), spouted bed drying (SB), air circulation oven drying at 60 °C (O60), and (D) air circulation oven drying at 105 °C (O105).

Table 5 summarizes this information and reinforces the structural changes resulting from oven drying. Lignins L and SB exhibited higher signal area values for protons in the aliphatic region compared to lignin O60 (0.58 and 0.86 versus 0.42). For lignin O105, the sample showed lower solubility in DMSO; this may explain the broad, low-intensity signals in the aliphatic region, despite the larger signal area. Lignins L and SB displayed higher area values in the side-chain region (10.59 and 12.89), a finding consistent with the results from color, UV profile, and FTIR analyses. Notably, samples O60 and O105 exhibit significant signal overlap between the Hα signal (at 4.3 ppm) and the methoxy group protons (δ = 3.0–4.0 ppm). In a ^1^H study, Bacchus and Fatehi^43^ also observed a reduction in H_α_ and H_β_ signals in a lignin sample following oven drying. The decrease in the signal corresponding to the aldehyde group proton at 10.0 ppm serves as another indicator of the severity of oven drying.

**Table 5:** Integrated Area Values in the ^1^H NMR Spectra Profiles of Corn Cob Lignins Dried by Freeze-Drying (L), Spouted Bed (SB), and Air Circulation Oven at 60 °C (O60) and 105 °C (O105)

Functional groups	δ range	L	SB	O60	O105
Aliphatic region	0.5–2.0	0.58	0.86	0.42	0.83
Side-chain region	4.0–6.0	10.59	12.39	8.53	9.89
Aromatic and vinyl region	6.0–8.0	3.55	4.57	4.5	2.79
Phenolic hydroxyls and aldehyde region	8.0–10.0	0.22	0.26	0.16	-

In the ^13^C NMR analysis, all samples generally exhibited the same signals corresponding to the aliphatic (∼40.0 ppm) and oxygenated aliphatic (66–77 ppm) regions, as shown in Figure 7B. Signals from the anomeric carbon of the lignin-carbohydrate complex (102.2 ppm) and from aromatic moieties were also observed in the samples (such as at 116.5 ppm for C-5 of the G unit and 130.6 ppm for C-1 of the nonetherified 5–5 unit).^44,45^ Under more severe stress, the O105 spectrum appeared even simpler, lacking the p-coumarate (PCE) signals at 144.7, 160.0, and 168.4 ppm, which correspond to C-7, C-4, and C-9.^45^ PCE is linked to the lignin side chain via a γ-ester bond and is a highly labile group.^46^

### 3.6 SPF of Moisturizing Cream Blended with Dried Lignins

Adding lignin to the moisturizing cream significantly reduced transmittance in the UV region, as shown in Figure 8. While the moisturizing cream sample exhibited behavior characteristic of a logarithmic function, the blend transmittance spectra showed two plateaus, with the lower-intensity one appearing in the UVB region (290–320 nm). As discussed in Section 3.4, double bonds facilitate radiation absorption; this also explains the even lower transmittance values observed in this range when lignin content increased from 1% to 2% (w/w). Studies such as those by Qian et al.^47^ and Van Duy et al.^48^ also reported that lignin content in blends directly influences UV-blocking capacity.

**Figure 8:**
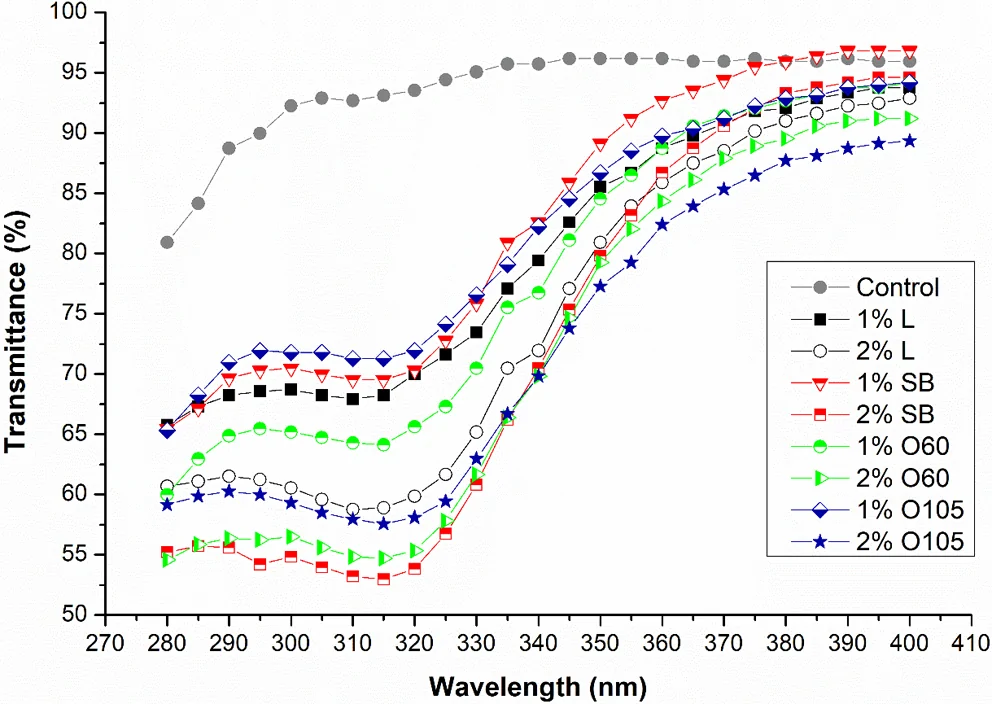
Transmittance of moisturizing cream blends with corn cob lignins obtained from freeze-drying (L), spouted bed drying (SB), and oven drying at 60 °C (O60) and 105 °C (O105). Blends were prepared with lignin dosages of 1% and 2% (w/w).

Regarding the best drying method, the discussion is less straightforward. In general, lignins obtained via oven drying showed higher SPF values than lignins L and SB, with lignin O60 standing out (Table 6). At the 2% (w/w) O60 concentration, the cosmetic blend achieved an SPF of 1.39, representing a 30.6% increase over the moisturizing cream without lignin (1.07). However, the SPF calculation considers the entire UV radiation range, and the blend ranking would shift if the UVB and UVA regions were evaluated separately. For instance, the blend with 2% (w/w) lignin SB showed the lowest transmittance values in the UVB region. Still, it exhibited a sharp rise in the UVA region, with values generally exceeding those of blends containing 1% (w/w) lignin. Conversely, the blend with 2% (w/w) lignin O105 showed only the third-lowest transmittance value in the UVB region; however, its spectrum rose slightly in the UVA region without exceeding 90% transmittance. This behavior in the lignin blends likely reflects a balance between phenolic hydroxyl content and structural packing. Meanwhile, blends containing lignins L and SB exhibited a lighter color (Figure S2) and align with researchers’ interest in lignins compatible with current sunscreen products. Lignins O60 and O105 produced darker-colored blends, but they could serve as ingredients in cosmetic formulations for individuals with darker skin tones. Thus, the results demonstrate that the choice of drying method must be strategic for the potential industrial use of lignin as a sunscreen ingredient. Unlike searching for new lignin sources or functionalization methods involving toxic chemicals, drying can modulate the characteristics of a single lignin type as needed, potentially simplifying the entire production chain.

**Table 6:** SPF Values and Boost Factor for Lignin Blends and Moisturizing Cream^a^

Sample	Control	1% L	2% L	1% SB	2% SB	1% O60	2% O60	1% 0105	2% O105
SPF/Increasing factor (%)	1.07^e^	1.25^cd^/17.3	1.34^abc^/25.5	1.21^d^/13.7	1.38^ab^/29.3	1.28^bcd^/20.1	1.39^a^/30.6	1.23^d^/14.9	1.39^ab^/30.0

a Lowercase letters indicate no statistical difference between the parameters at the *p* < 0.05 level.

The SPF tests were designed to evaluate the effects of each drying method; therefore, we selected a cosmetic product that did not contain a sunscreen (NIVEA Soft moisturizing cream). Structural changes induced by drying, combined with potential synergism with a pre-existing sunscreen, would have made the system overly complex to interpret and could have distracted from the study’s primary objective. Nevertheless, incorporating lignins into cosmetic formulations that already contain sunscreens (whether commercial products or simulated ones) could be a topic for future research, given that authors such as Qian et al.^49^ and Fidelis et al.^50^ have noted that this macromolecule can boost SPF. Similarly, it would be interesting to investigate the potential effects on skin health from adding different lignin samples.

### 3.7 Antioxidants and Antibacterial Activity of Lignins Dried by Different Methods

Sigmoidal trends can be observed in antioxidant activity tests, but each test showed particular behavior (Figure 9). Lignins obtained by oven drying (O60 and O105) showed better DPPH radical-scavenging potential, reaching a maximum of 80.8% at 0.21 mg/L. Lignins L and SB tended to stabilize even before reaching the 50% scavenging value. In contrast, lignins L and SB showed slightly superior antioxidant activity in the ABTS tests, with an IC_50_ of approximately 0.012 mg/L, while the others showed approximately 0.02 mg/L.

**Figure 9:**
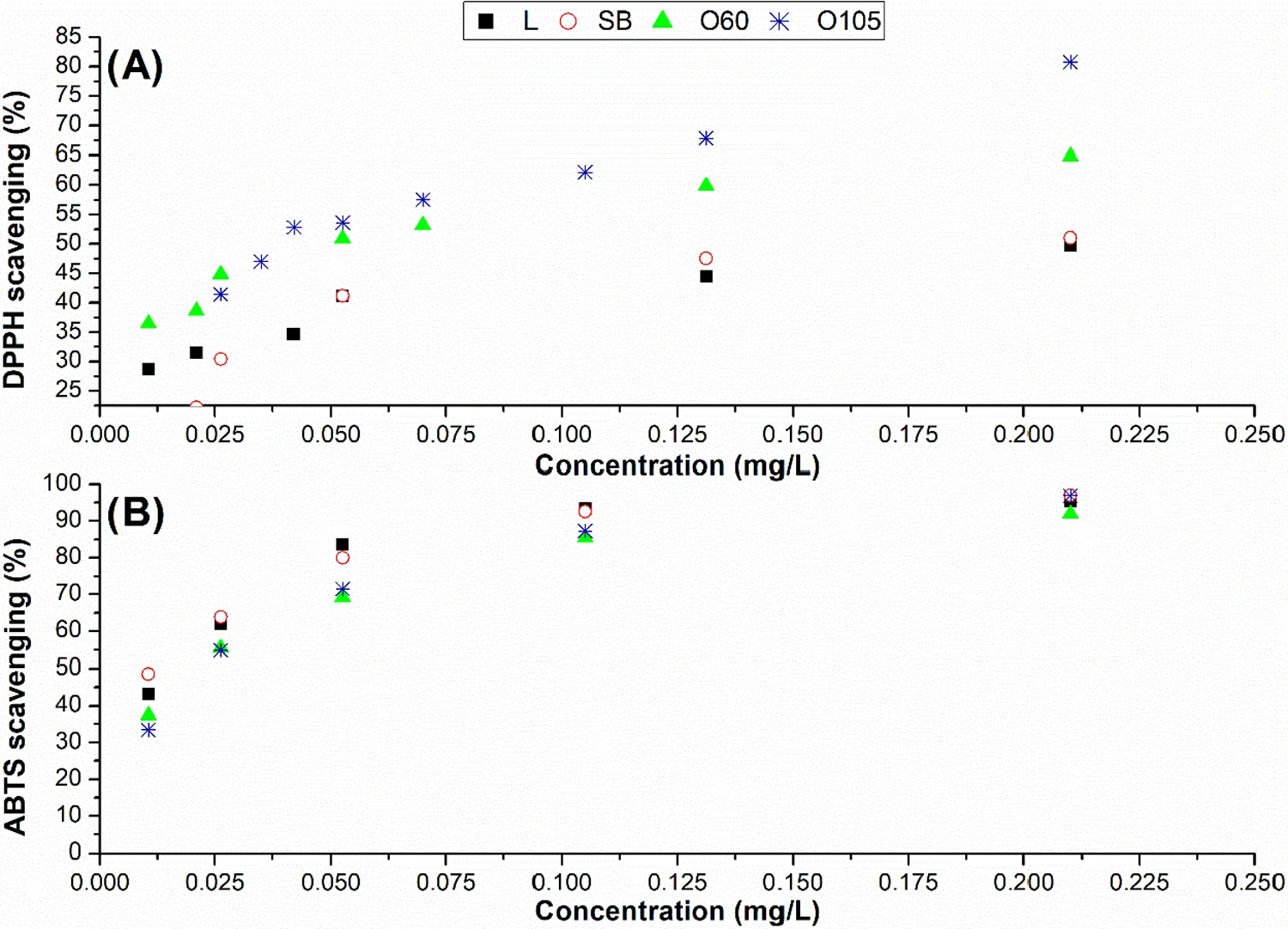
Results of DPPH (A) and ABTS (B) radical scavenging after contact with ethanolic solutions of lignins from freeze-drying (L), spouted bed drying (SB), and oven drying at 60 °C (O60) and 105 °C (O105).

The abundant availability of phenylpropanoids in lignin enables proton or electron donation to stabilize free radicals via mechanisms known as hydrogen atom transfer (HAT) and single electron transfer (SET). HAT and SET govern antioxidant activity, with the phenolic hydroxyl group playing a critical role.^51^ While the ABTS^•+^ radical cation is highly delocalized and accessible to lignins (particularly samples L and SB, which exhibit higher phenolic hydroxyl content), the unpaired electron of the DPPH radical is surrounded by two phenyl rings and a picryl ring; these bulky groups create steric hindrance.^52^ Consequently, it is logical that lignins O60 and O105 (which possess shorter side chains) demonstrate superior DPPH radical inhibition, creating a discrepancy between performance and the lignins’ phenolic hydroxyl content or theoretical potential. This is also one reason DPPH radical assays are less preferred than ABTS assays for evaluating the antioxidant activity of lignins.

Table 7 shows the occurrence of antimicrobial activity and the average inhibition zone values for all corn cob lignin samples. Tests using only DMSO (control) showed no effects on the strains, ruling out any influence on the analysis. Although some studies have reported the efficacy of lignins against Gram-negative bacteria,^37,38^ none of the lignin samples in the present study affected the *Pseudomonas aeruginosa* strain (Figure S3). Literature indicates that the lipopolysaccharide (LPS) on the outer surface of these bacteria interacts poorly with unmodified lignins, given their contrasting chemical natures (hydrophilic vs hydrophobic) and the fact that both the LPS and the lignin macromolecules carry a net negative charge.^53,54^ Consequently, the loss of hydrophilic groups and the resulting structural compaction of the lignin during extreme drying conditions run counter to the strategies required to achieve effective performance against Gram-negative bacteria.

**Table 7:** Zone of Inhibition (mm) of L, SB, O60 and O105 against Strains of *Pseudomonas aeruginosa* and *Rhodococcus rhodochrous* ^a^

Samples	*Pseudomonas aeruginosa* ATCC 27853 (Gram-negative)	*Rhodococcus rhodochrous* NRRL B–2149 (Gram-positive)
Only DMSO (Control)	-	-
L	-	+ (0.91 ± 0.12^c^)
SB	-	+ (1.92 ± 0.12^a^)
O60	-	+ (1.00 ± 0.00^b^)
O105	-	+ (0.88 ± 0.13^c^)

a Lowercase letters indicate no statistical difference between the parameters at the *p* < 0.05 level.

In contrast, all lignins inhibited growth of *Rhodococcus rhodochrous*, a Gram-positive bacterium. Gram-positive bacteria are more susceptible to collapse due to a weaker barrier effect; consequently, lignins can disrupt the plasma membrane, inhibit the respiratory chain, and promote the accumulation of reactive species in the cytoplasm.^55^ These inhibition mechanisms are also linked to phenolic hydroxyl content, which helps explain why SB lignin yielded the best result (1.92); however, L lignin performed as well as the oven-dried lignins. Because inhibition is multifactorial, involving mechanical and biochemical aspects related to adhesion and cell rupture, a more in-depth investigation of lignin characteristics may be needed to explain these results. Another possibility would be to switch from agar plate cultures to liquid media cultures. Although the agar-based approach was successful, the semisolid medium limits lignin mobility; switching to liquid media could improve the accessibility of lignins (in micro- or nanoparticle form) to the cells and allow determination of a more precise metric, such as the minimum inhibitory concentration (MIC).

### 3.8 Acute Lignin Toxicity Test in Zebrafish

Given the known antimicrobial activity of natural phenolic compounds and the acute toxicity described for some phenolic derivatives,^39,40^ perhaps the lignin samples could induce adverse effects in zebrafish embryos. However, this hypothesis was not confirmed under the experimental conditions evaluated. Lignin samples did not affect embryo survival throughout the 96 h of exposure, even at the highest concentrations tested (Figure 10). Survival percentages in the lignin-exposed groups ranged from 92.5% to 95.0%, similar to those in the negative control. Stine et al.^56^ observed that uncoated lignin nanoparticles did not significantly increase zebrafish embryo mortality up to the highest concentrations evaluated. In contrast, chitosan-coated lignin nanoparticles showed toxicity only at high concentrations, with an LC_50_ of 105 mg/L in embryos without chorion and 548 mg/L in embryos with chorion. Using lignin-wheat stalk carbohydrate complexes, Gu et al.^57^ reported near 100% survival between 0.01 and 10 mg/L and only a slight reduction of approximately 5% in survival when the concentration increased from 10 to 100 mg/L, indicating an absence of relevant toxicity below 100 mg/L.

**Figure 10:**
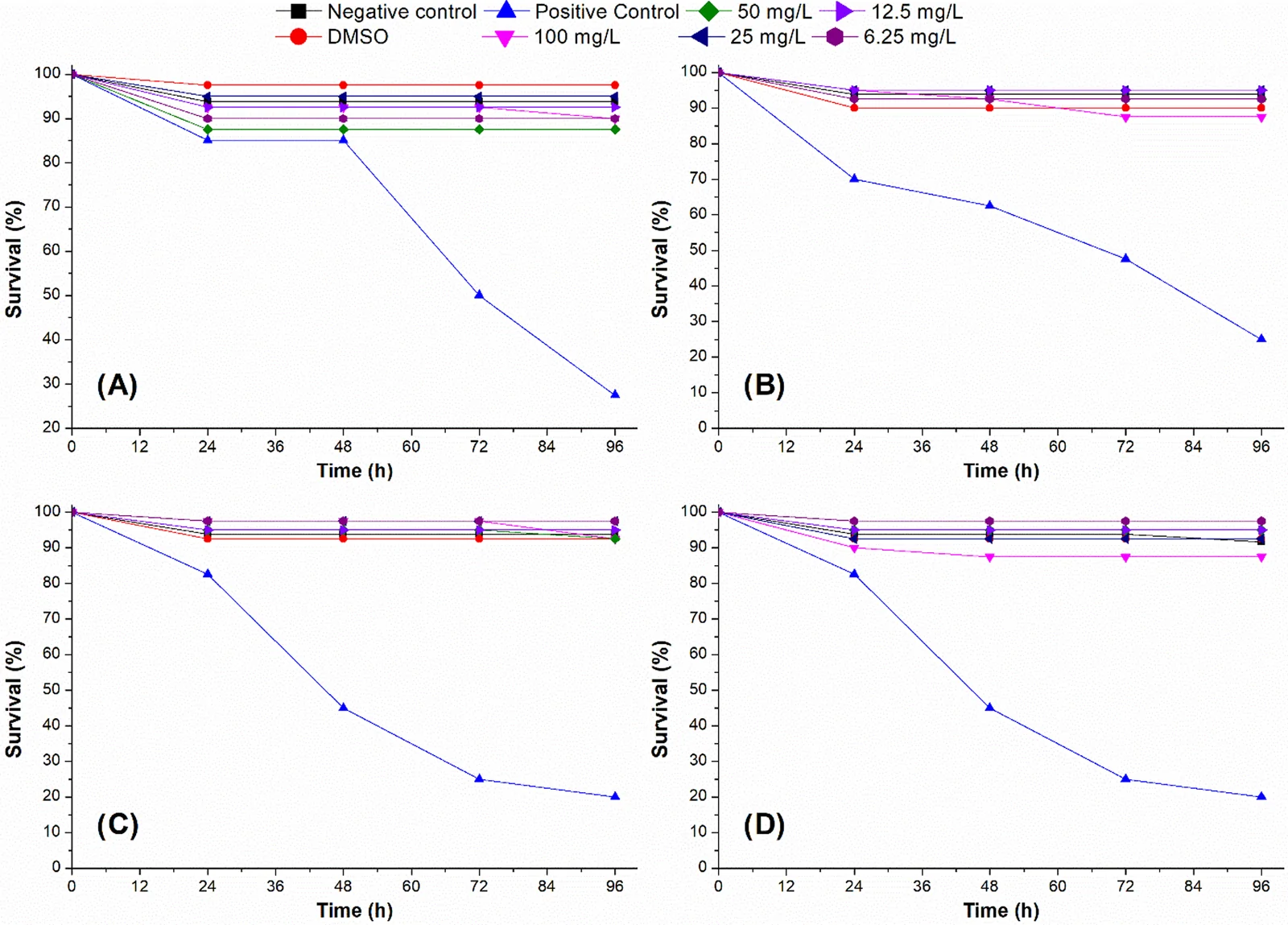
Survival rates of zebrafish incubated with corn cob lignins dried by freeze-drying (L-A), spouted bed (SB-B), and air-circulation oven at 60 °C (O60–C) and 105 °C (O105-D).

Hatching is an informative complementary parameter in the FET assay, as changes in this process can reflect disturbances in embryonic development, even without excessive lethality. Accordingly, OECD Guideline 236 recommends recording hatching rates in both treatment and control groups as part of the assay interpretation and reporting.^58^ In the present study, the temporal distribution of hatching did not differ significantly between the controls (water and 0.1% DMSO) and the tested concentrations of L (*p* = 0.936), SB (*p* = 0.335), O60 (*p* = 0.436), or O105 (*p* = 0.737; Fisher’s exact test) (Table S1). Morphological abnormalities were uncommon and occurred as isolated observations, showing no apparent concentration-dependent pattern. In the L group, one embryo with caudal malformation was observed at 100 mg/L, and another at 12.5 mg/L. In the O105 group, two affected embryos were observed at 100 mg/L: one with caudal malformation and pericardial edema, and the other with caudal malformation only. No morphological abnormalities were observed in the other lignin-treated groups. Due to the low number of malformations and the predominance of zero counts across treatments, morphological abnormalities were described summarily and were not subjected to statistical analysis. Bragato et al.,^59^ for example, reported morphological abnormalities in less than 4% of exposed zebrafish embryos, whereas Stine et al.^56^ observed developmental alterations primarily at substantially higher exposure levels. Thus, the occasional abnormalities observed in this study are best interpreted as a low-frequency occurrence rather than evidence of a reproducible teratogenic response.

These results demonstrate preliminary safety for using lignin as an ingredient in cosmetics or other derived products. Furthermore, the data suggest that the different synthesis, drying, and purification conditions evaluated did not generate byproducts or physicochemical modifications capable of generating detectable lethal or teratogenic effects in zebrafish embryos at the tested concentrations.

## 4 Conclusions

This study shows that drying techniques can strongly affect lignin structure–property relationships. Conventional oven drying induced condensation reactions and significantly increased UV-blocking performance, while spouted bed drying better preserved color and phenolic hydroxyl content, with fewer changes to aggregate morphology. Tests on zebrafish embryos confirmed the compatibility and nontoxic nature of the lignins obtained in all drying processes. Overall, no significant differences were observed between the characteristics of lignins L and SB. Even so, spouted bed drying cannot be viewed as a panacea, as this method yielded very poor product recovery. The difficulties encountered during SB operation (interactions between lignin and HDPE spheres and cyclone losses), if not resolved, pose prohibitive barriers to process scalability, suggesting that the method may ultimately remain limited to the laboratory scale. These findings reinforce the importance of paying attention to all stages of technical lignin preparation, since it can be a fundamental raw material or ingredient for the transition to a circular bioeconomy.

## Supplementary Material



## Notes

Correlation plots between condensation index (CI) and total hydroxyl content (A), and between CI and Δ*E*, obtained from lignin color assays (B) (Figure S1); lignins obtained via different drying techniques at various concentrations, evaluated for their synergistic effect with a moisturizing sunscreen cream; drying methods include freeze-drying (L), spouted bed drying (SB), air circulation oven drying at 60 °C (O60), and air circulation oven drying at 105 °C (O105), source: own authorship (Figure S2); disk diffusion assay for antimicrobial activity assessment, evaluation of bacterial growth inhibition under different treatments in agar plates inoculated with (A) *Rhodococcus rhodochrous* and (B) *Pseudomonas aeruginosa*, the blue lines delimit the quadrants corresponding to the different lignins tested (Figure S3); cumulative hatching rates of zebrafish embryos exposed to different lignin samples, data are presented as *n*/*N* (%), where *n* is the cumulative number of hatched embryos and *N* is the number of embryos capable of hatching, excluding those that coagulated within the first 24 h, hatching was assessed at 48, 72, and 96 hpf (Table S1) (PDF)
