# Supplementary material for: Effects of Drying on the Properties and Applications of Alkaline Corn Cob Lignin
Source: ACS Omega. 2026 Oct 17;11(43):66845–57. doi: 10.1021/acsomega.6c06720 (PMC13737891; doi:10.1021/acsomega.6c06720)
Supplement: Supplementary file 1 [file ao6c06720_si_001.pdf]

EFFECTS OF DRYING ON THE PROPERTIES AND APPLICATIONS OF ALKALINE CORN COB LIGNIN

Carlos Eduardo de Araújo Padilha[1]; Jadna Lúcia de Freitas Silva[1]; Francisco Bruno Ferreira de Freitas[2]; Gleyson Batista de Oliveira[1]; Augusto Monteiro de Souza[3]; Darlisson de Alexandria Santos[4]; Thelma Sley Pacheco Cellet[5]; Renata Martins Braga[6]; Emmanuel Damilano Dutra[4]; Jackson Araújo de Oliveira[1]; Domingos Fabiano de Santana Souza[1].*

[1]Laboratory of Alternative Energy and Transport Phenomena, Chemical Engineering Department, Federal University of Rio Grande do Norte (UFRN), Natal-RN, Brazil.

[2]Laboratory of Biochemical Engineering, Chemical Engineering Department, Federal University of Rio Grande do Norte (UFRN), Natal-RN, Brazil.

[3]Department of Cell Biology and Genetics, Biosciences Center, Federal University of Rio Grande do Norte (UFRN), Natal-RN, Brazil.

[4]Research Group on Biomass Energy, Department of Nuclear Energy, Federal University of Pernambuco (UFPE), Recife-PE, Brazil.

[5]Polymeric and Composite Materials Group, Department of Chemistry, State University of Maringá (UEM), Maringá-PR, Brazil.

[6]Enviromental Technology Laboratory, Department of Chemistry, Federal University of Rio Grande do Norte (UFRN), Natal-RN, Brazil.

Fig. S1: Correlation plots between Condensation Index (CI) and total hydroxyl content (A), and between CI and ΔE, obtained from lignin color assays (B).

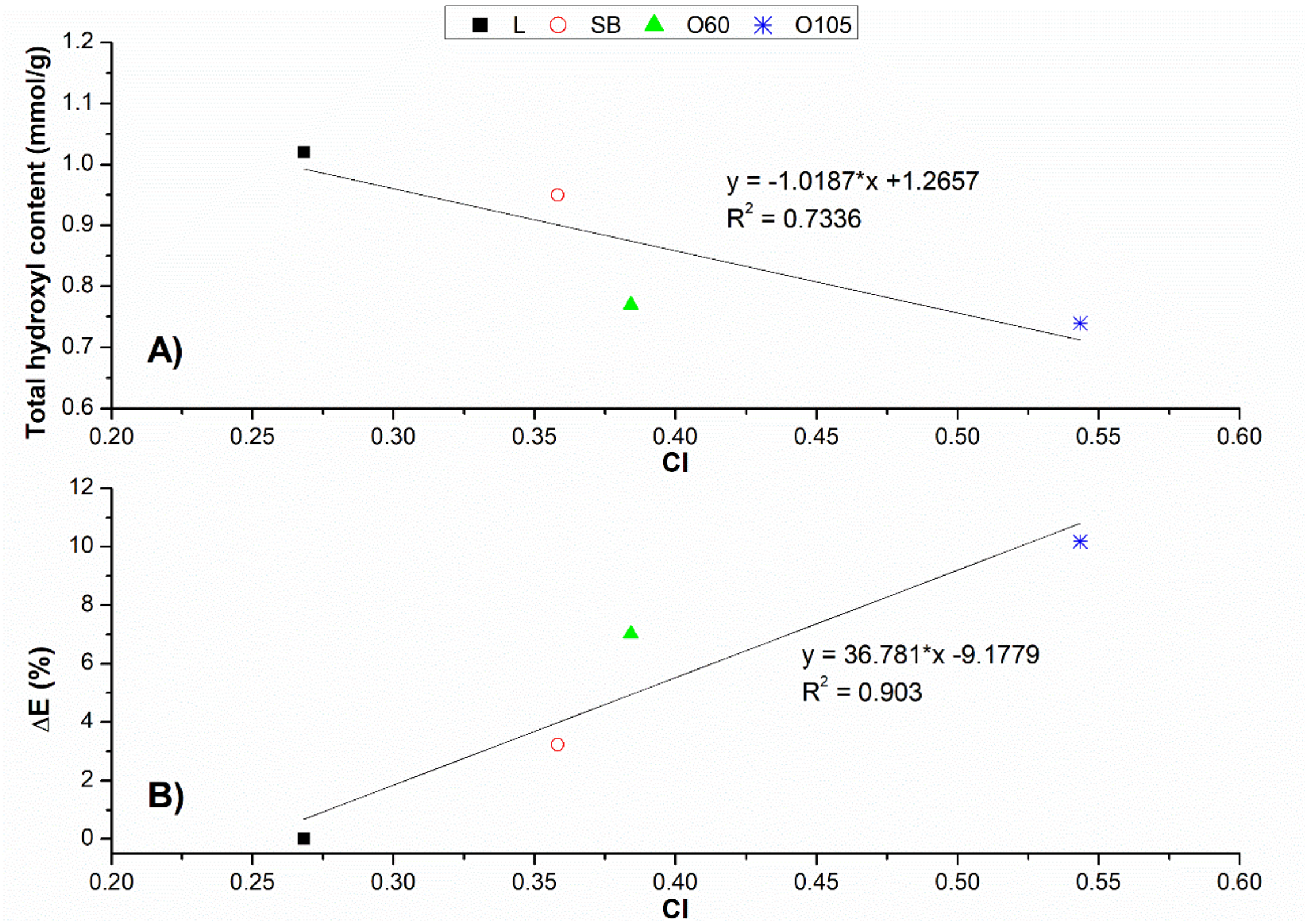

Fig. S2: Lignins obtained via different drying techniques at various concentrations, evaluated for their synergistic effect with a moisturizing sunscreen cream. Drying methods include freeze-drying (L), spouted bed drying (SB), air circulation oven drying at 60 °C (O60), and air circulation oven drying at 105 °C (O105). Source: Own authorship

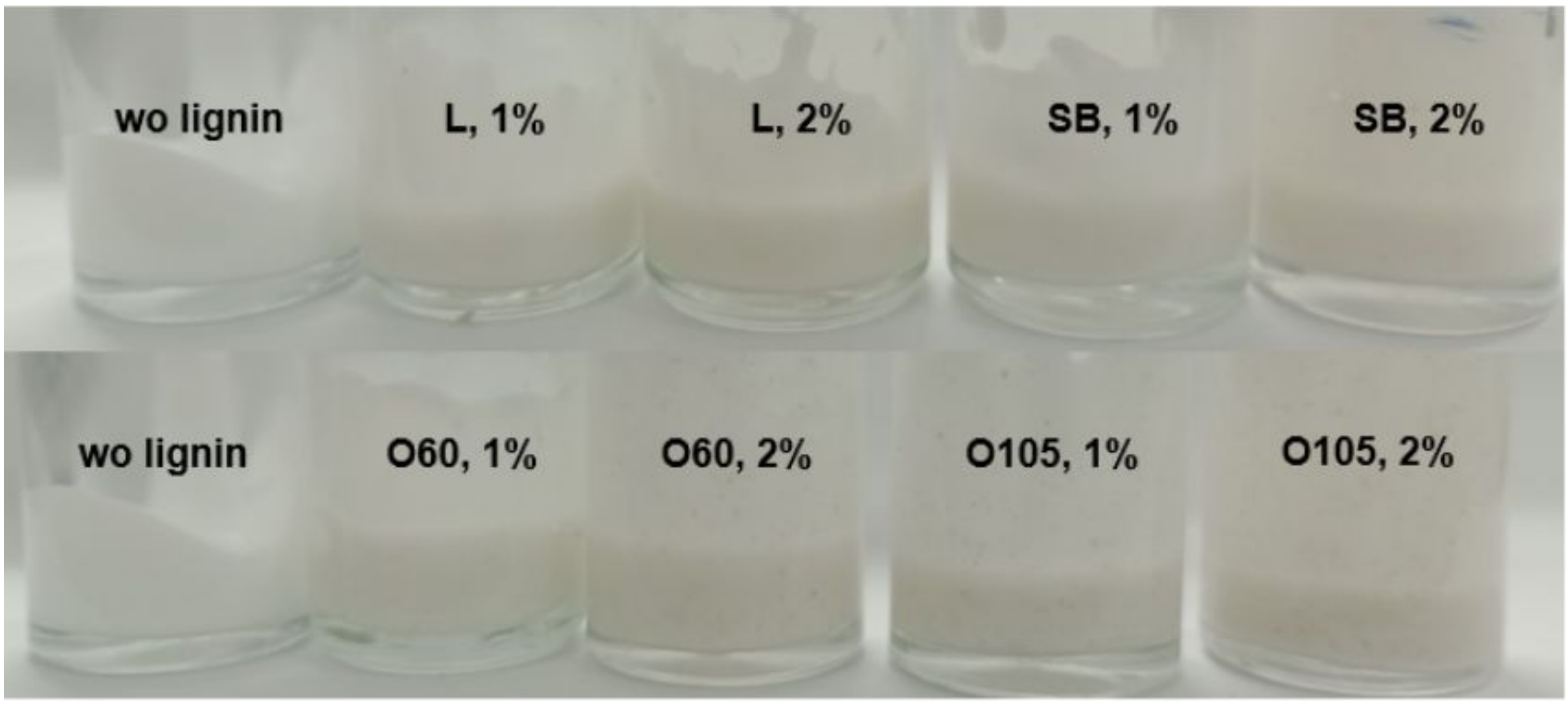

Fig. S3: Disk diffusion assay for antimicrobial activity assessment. Evaluation of bacterial growth inhibition under different treatments in agar plates inoculated with (A) *Rhodococcus rhodochrous* and (B) *Pseudomonas aeruginosa*. The blue lines delimit the quadrants corresponding to the different lignins tested. Source: Own authorship

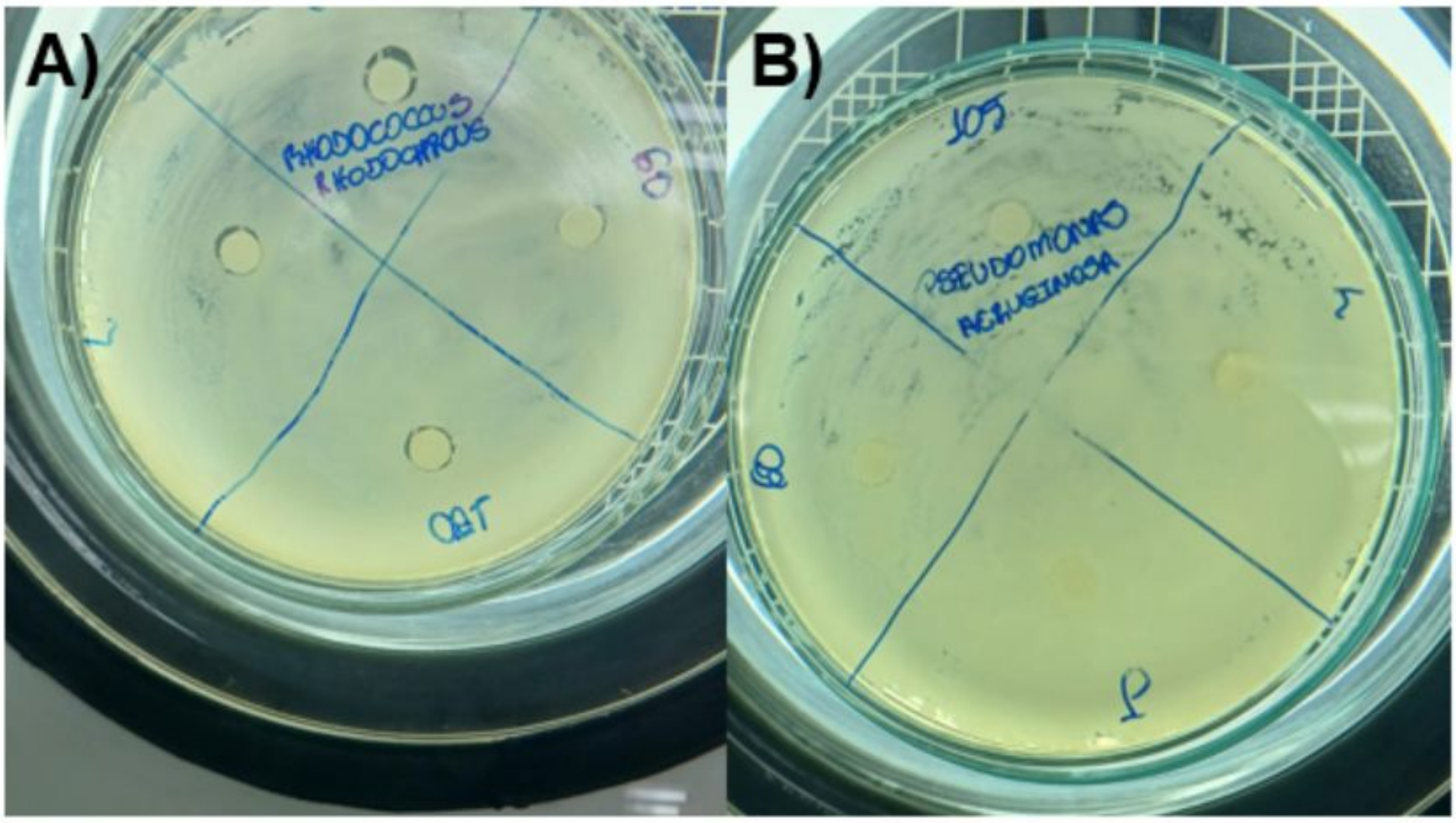

Table S1. Cumulative hatching rates of zebrafish embryos exposed to different lignin samples. Data are presented as n/N (%), where n is the cumulative number of hatched embryos and N is the number of embryos capable of hatching, excluding those that coagulated within the first 24 h. Hatching was assessed at 48, 72, and 96 hpf.

**Lignin**	**Treatment**	**Eligible embryos (N)**	**48 hpf, n/N (%)**	**72 hpf, n/N (%)**	**96 hpf, n/N (%)**
**L**	Control -	45	3/45 (6.7)	30/45 (66.7)	45/45 (100.0)
	Control (DMSO 0.1%)	39	4/39 (10.3)	32/39 (82.1)	39/39 (100.0)
	Control +	34	0/34 (0.0)	6/34 (17.6)	14/34 (41.2)
	100 µg/mL	37	5/37 (13.5)	28/37 (75.7)	37/37 (100.0)
	50 µg/mL	35	5/35 (14.3)	26/35 (74.3)	35/35 (100.0)
	25 µg/mL	38	3/38 (7.9)	31/38 (81.6)	38/38 (100.0)
	12.5 µg/mL	37	2/37 (5.4)	28/37 (75.7)	37/37 (100.0)
	6.25 µg/mL	36	4/36 (11.1)	30/36 (83.3)	36/36 (100.0)
**SB**	Control -	45	3/45 (6.7)	30/45 (66.7)	45/45 (100.0)
	Control (DMSO 0.1%)	36	0/36 (0.0)	28/36 (77.8)	36/36 (100.0)
	Control +	28	0/28 (0.0)	5/28 (17.9)	10/28 (35.7)
	100 µg/mL	38	2/38 (5.3)	30/38 (78.9)	38/38 (100.0)
	50 µg/mL	37	1/37 (2.7)	26/37 (70.3)	37/37 (100.0)
	25 µg/mL	38	4/38 (10.5)	27/38 (71.1)	38/38 (100.0)
	12.5 µg/mL	38	2/38 (5.3)	30/38 (78.9)	38/38 (100.0)
	6.25 µg/mL	37	1/37 (2.7)	33/37 (89.2)	37/37 (100.0)
**O60**	Control -	45	6/45 (13.3)	32/45 (71.1)	45/45 (100.0)
	Control (DMSO 0.1%)	37	3/37 (8.1)	34/37 (91.9)	37/37 (100.0)
	Control +	33	0/33 (0.0)	0/33 (0.0)	10/33 (30.3)
	100 µg/mL	39	4/39 (10.3)	30/39 (76.9)	39/39 (100.0)
	50 µg/mL	38	3/38 (7.9)	31/38 (81.6)	38/38 (100.0)
	25 µg/mL	39	5/39 (12.8)	34/39 (87.2)	39/39 (100.0)
	12.5 µg/mL	39	6/39 (15.4)	30/39 (76.9)	39/39 (100.0)
	6.25 µg/mL	39	3/39 (7.7)	36/39 (92.3)	39/39 (100.0)
**O105**	Control -	45	6/45 (13.3)	32/45 (71.1)	45/45 (100.0)
	Control (DMSO 0.1%)	37	3/37 (8.1)	34/37 (91.9)	37/37 (100.0)
	Control +	33	0/33 (0.0)	0/33 (0.0)	10/33 (30.3)
	100 µg/mL	36	3/36 (8.3)	28/36 (77.8)	36/36 (100.0)
	50 µg/mL	38	4/38 (10.5)	29/38 (76.3)	38/38 (100.0)
	25 µg/mL	37	4/37 (10.8)	30/37 (81.1)	37/37 (100.0)
	12.5 µg/mL	38	3/38 (7.9)	34/38 (89.5)	38/38 (100.0)
	6.25 µg/mL	39	5/39 (12.8)	34/39 (87.2)	39/39 (100.0)
